# Cognitive function and brain plasticity in a rat model of shift work: role of daily rhythms, sleep and glucocorticoids

**DOI:** 10.1038/s41598-020-69969-x

**Published:** 2020-08-04

**Authors:** Andrea R. Marti, Torhild T. Pedersen, Jonathan P. Wisor, Jelena Mrdalj, Øystein Holmelid, Sudarshan Patil, Peter Meerlo, Clive R. Bramham, Janne Grønli

**Affiliations:** 1 Bergen Stress and Sleep Group, Department of Biological and Medical Psychology, Faculty of Psychology, University of Bergen, Jonas Liesvei 91, 5009 Bergen, Norway; 2 College of Medicine, Washington State University, Spokane, WA USA; 3 Department of Biomedicine, Faculty of Medicine, University of Bergen, Bergen, Norway; 4 Groningen Institute for Evolutionary Life Sciences, University of Groningen, Groningen, The Netherlands; 5 Present Address: Bergen Stress and Sleep Group, Department of Biological and Medical Psychology, Faculty of Psychology, University of Bergen, Jonas Liesvei 91, 5009 Bergen, Norway

**Keywords:** Circadian regulation, Sleep, Wakefulness

## Abstract

Many occupations require operations during the night-time when the internal circadian clock promotes sleep, in many cases resulting in impairments in cognitive performance and brain functioning. Here, we use a rat model to attempt to identify the biological mechanisms underlying such impaired performance. Rats were exposed to forced activity, either in their rest-phase (simulating night-shift work; rest work) or in their active-phase (simulating day-shift work; active work). Sleep, wakefulness and body temperature rhythm were monitored throughout. Following three work shifts, spatial memory performance was tested on the Morris Water Maze task. After 4 weeks washout, the work protocol was repeated, and blood and brain tissue collected. Simulated night-shift work impaired spatial memory and altered biochemical markers of cerebral cortical protein synthesis. Measures of daily rhythm strength were blunted, and sleep drive increased. Individual variation in the data suggested differences in shift work tolerance. Hierarchical regression analyses revealed that type of work, changes in daily rhythmicity and changes in sleep drive predict spatial memory performance and expression of brain protein synthesis regulators. Moreover, serum corticosterone levels predicted expression of brain protein synthesis regulators. These findings open new research avenues into the biological mechanisms that underlie individual variation in shift work tolerance.

## Introduction

We live in a 24-h society, where services are required around the clock. For this reason, a significant proportion of the work force is required to work shifts. The temporal aspects of the work schedule, night shift work in particular, has negative consequences for cognitive functioning. The risk of errors and accidents is increased on the night shift compared to morning and afternoon shifts, and the risk increases across consecutive night shifts worked^1,2^.

It has long been assumed that the cause for cognitive dysfunction on the night shift is lack of sleep, and the link between perturbed sleep and cognitive dysfunction is well documented^3,4^. In the case of night shift work, both homeostatic and circadian aspects of sleep are challenged. Workers are required to stay awake for prolonged hours at times of day when the circadian system promotes rest, and to sleep at times when the circadian system promotes wakefulness^5,6^.

In addition to disturbed sleep and circadian rhythm regulation, night shift work can act as a stressor on bodily systems, which may result in adverse health effects. Both acute sleep deprivation and chronic sleep restriction are known to induce neuroendocrine stress, as evidenced by elevated activity in the hypothalamic–pituitary–adrenal (HPA) axis, resulting in increased levels of circulating corticosteroids^7^. Night shift work has been shown to alter the daily rhythm of circulating corticosteroids in both laboratory and field studies^8–10^.

Disturbances in circadian organization not only disrupt sleep and circulating corticosteroids, but also many other biological mechanisms, including brain de novo protein synthesis^11,12^. In order to maintain the neurobiological substrates of cognitive functioning including synaptic plasticity, the brain is required to constantly synthesize new proteins. The rate of protein synthesis is commonly regulated at the level of initiation of translation^13,14^. This process depends on binding of eukaryotic initiation factor 4E (eIF4E) to the mRNA 5′-cap, and recruitment of several factors to form the translation initiation complex eIF4F^15^. Translation initiation is also in part promoted by the circadian clock protein brain-and-muscle arnt-like protein 1 (BMAL1)^16^.

Utilizing a rat model of shift work, we recently showed that 3–4 consecutive simulated night shifts induced deficits in waking function, with modest effects on sleep time^17^. Moreover, we identified impairments to BMAL1-driven translational activity in the prefrontal cortex (PFC) of rats following three days of simulated night shift work^11^.

While the negative effects of working night shifts are robust at the group level, there are considerable individual differences in tolerance to shift work. Shift work tolerance, the ability to adapt to shift work without adverse consequences, has been linked to a number of traits relating to age, gender, chronotype and personality^18^. Workers who self-report low shift work tolerance do not show objectively shorter or poorer sleep than those with high tolerance, but they do self-report less efficient sleep and show elevated levels of sleepiness and impaired cognitive functioning^19^. Identification of biological markers or predictors of shift work tolerance are lacking.

The biological mechanisms that underlie the negative effects of night shift work on cognitive performance and brain functioning remain unknown. In the present study we show, using a rat model, that markers of daily rhythmicity, sleep drive and consolidation of sleep, and circulating corticosteroids all predict different aspects of the negative impact of shift work on brain functioning and cognitive performance.

## Results

### Rest work impairs daily rhythmicity

**Figure 1:**
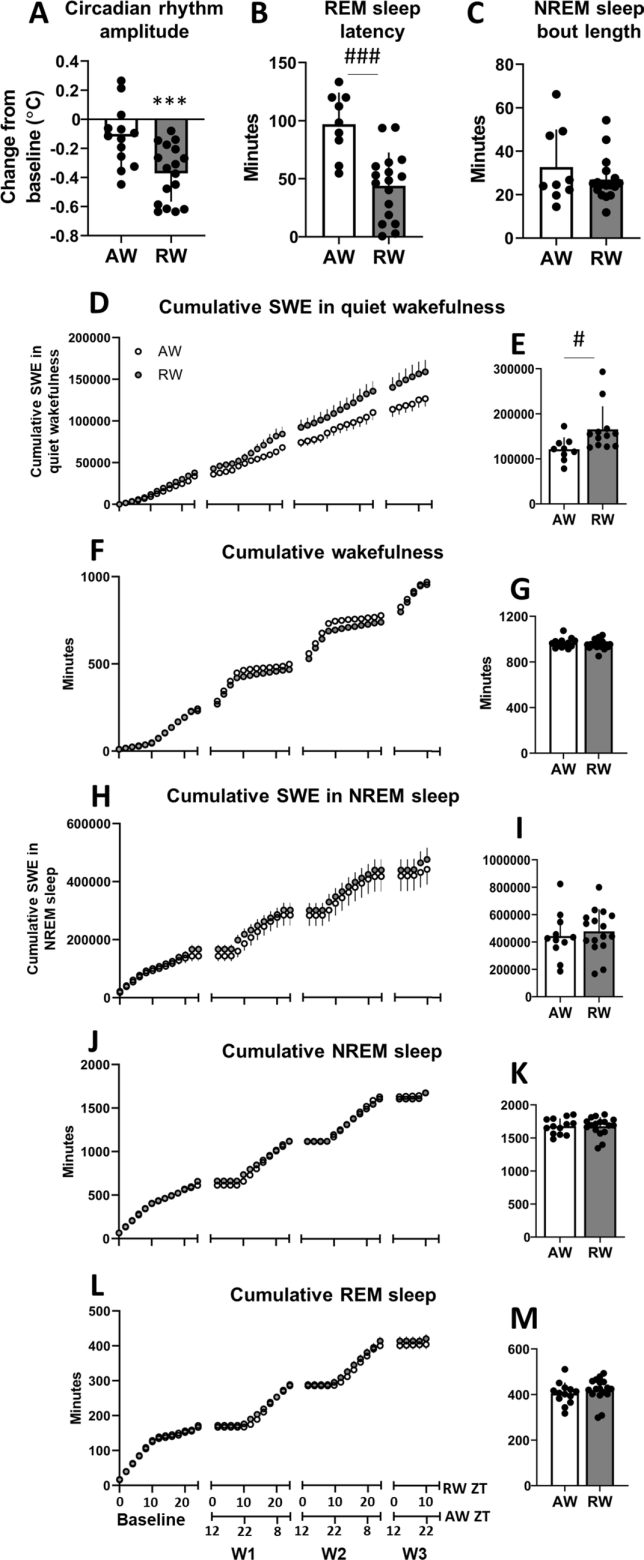
Daily rhythm, sleep and wake parameters in active workers (AW) and rest workers (RW). (**A**) Body temperature rhythm amplitude during the 3-day work schedule relative to baseline. Body temperature rhythm was estimated with cosinor analysis. (**B**) Latency to rapid eye movement (REM) sleep after the 2nd work shift. (**C**) Mean length of non-REM (NREM) sleep bouts during the 3-day work period. (**D**,**H**) Cumulative slow wave energy (SWE, total integrated power in the 1–4 Hz frequency range) in quiet wakefulness and NREM sleep. (**F**,**J**,**L**) Cumulative wakefulness, NREM sleep and REM sleep, respectively. All cumulative data are expressed per 2 h during baseline and 3-day work schedule; workday 1 (W1) to W3 in RW (work hours: zeitgeber time, ZT2-10) and AW (work hours: ZT14-22). (**E**,**I**) Total SWE in quiet wakefulness and NREM sleep, respectively, in AW and RW during the 3-day work period. (**G**,**K**,**M**) Total time in wakefulness, NREM sleep and REM sleep, respectively, in AW and RW during the 3-day work period. Bar plots show mean ± SEM, with scatter plot overlaid. N = 9–17/group. ***p < 0.001, compared to baseline. ^#^p < 0.05, ^###^p < 0.001, between groups.

During the 24-h baseline, body temperature rhythm amplitude did not significantly differ between groups (active worker, AW, mean 0.64 ± 0.11 °C versus rest worker, RW, mean 0.63 ± 0.13 °C; t_(28)_ = 0.27, p = 0.79, d = 0.10). The 3-day simulated night shift work (rest work) protocol impaired rhythm amplitude. Compared to baseline, rest workers exhibited a robust decrease in the rhythm amplitude by a mean of approximately 0.4 °C (t_(16)_ = 7.88, p < 0.001, d = -2.82; Fig. 1A), while the simulated day shift work (active work) protocol did not significantly alter the amplitude of body temperature rhythm, (t_(12)_ = 2.06, p = 0.062, d = − 0.73; Fig. 1A).

Since shift work can have differential effects on different bodily rhythms, rapid eye movement (REM) sleep latency was calculated as a sleep-related marker of daily rhythmicity^20^. Following the 2nd work shift, latency to enter REM sleep was significantly shorter following rest work than following active work (t_(24)_ = 4.56, p < 0.001, d = 1.90; Fig. 1B).

Importantly, individual differences in the rhythm parameters were considerable, with substantial overlap between the two work groups.

### Rest work enhances sleep drive, without effects on sleep time or intensity

Across the 24 h baseline condition, all animals showed nocturnal patterns of wakefulness and diurnal patterns of sleep. Wakefulness occurred primarily during dark phase (72.9 ± 3.8% during zeitgeber time, ZT12-24) and sleep during light phase (71.6 ± 5.6% during ZT0-12). During baseline, there were no significant differences between RW and AW in time spent in wakefulness (t_(28)_ = 0.49, p = 0.63, d = 0.18), NREM sleep (t_(28)_ = 0.63, p = 0.53, d = 0.24) or REM sleep (t_(28)_ = 0.44, p = 0.67, d = 0.16). No significant differences were found in the length of NREM sleep bouts (t_(28)_ = 1.26, p = 0.22, d = 0.46) or REM sleep bouts (t_(28)_ = 1.16, p = 0.26, d = 0.42).

As previously demonstrated^17^, forced activity for 8 h during the rest phase kept the rats awake except for sporadic micro-sleep episodes. Both groups spent more time awake and less time in sleep on work-days compared to baseline (AW wake t_(9)_ = 5.8, p < 0.001, d = 2.48, NREM t_(9)_ = 4.05, p = 0.003, d = 1.80, REM t_(9)_ = 4.9, p < 0.001, d = 2.18; RW wake t_(15)_ = 10.13, p < 0.001, d = 3.55, NREM t_(15)_ = 8.21, p < 0.001, d = 2.76, REM t_(15)_ = 6.80, p < 0.001, d = 1.72). Time spent in sleep during simulated shift work did not differ significantly between groups (t_(28)_ = 1.40, p = 0.17, d = 0.38). On average, RW spent 603 ± 53 and 610 ± 19 min (mean 606 ± 36 min), and AW spent 622 ± 41 and 624 ± 35 min (mean 623 ± 38 min) in sleep across the two successive working days. Rest work induced a redistribution of sleep to the endogenous active phase. During the undisturbed 16 h between the simulated work shifts, time in NREM sleep (t_(28)_ = 1.49, p = 0.15, d = 0.54) and REM sleep (t_(28)_ = 1.28, p = 0.21, d = 0.47) did not significantly differ between RW and AW. Following the 2nd work shift, latency to stable sleep (1 min of continuous sleep) was significantly shorter following rest work (13.8 ± 9.5 min) than following active work (32.1 ± 34.6 min) (t_(28)_ = 2.09, p = 0.046, d = 0.72).

Across the 3-day work period, groups did not significantly differ in the amount of cumulated wake time (t_(28)_ = 0.71, p = 0.49, d = 0.26; Fig. 1F,G), time spent in NREM sleep (t_(28)_ = 0.09, p = 0.93, d = 0.03; Fig. 1J,K) or REM sleep (t_(28)_ = 1.50, p = 0.15, d = 0.55, Fig. 1L,M), or accumulation of slow wave energy (1–4 Hz) during NREM sleep (t_(25)_ = 0.52, p = 0.61, d = 0.20, Fig. 1H,I). However, rest work led to higher accumulation of slow wave energy in quiet wakefulness, suggesting a degraded waking state and enhanced sleep drive (t_(19)_ = 2.26, p = 0.04, d = 1.04; Fig. 1D,E). These results replicate previous findings from our lab showing that work in the rest phase increases sleep drive, without affecting total time spent in sleep^17^.

Previous mathematical modelling of sleep data (combining two-process model and stochastic model) suggests that duration of NREM sleep bouts importantly contributes to the observed effects of simulated shift work on sleep/wake dynamics in our rodent model^21^. Here, the average length of NREM sleep bouts did not significantly differ between groups (t_(24)_ = 1.07, p = 0.30, d = 0.40; Fig. 1C), although individual differences were considerable.

### Rest work impairs spatial memory performance

**Figure 2:**
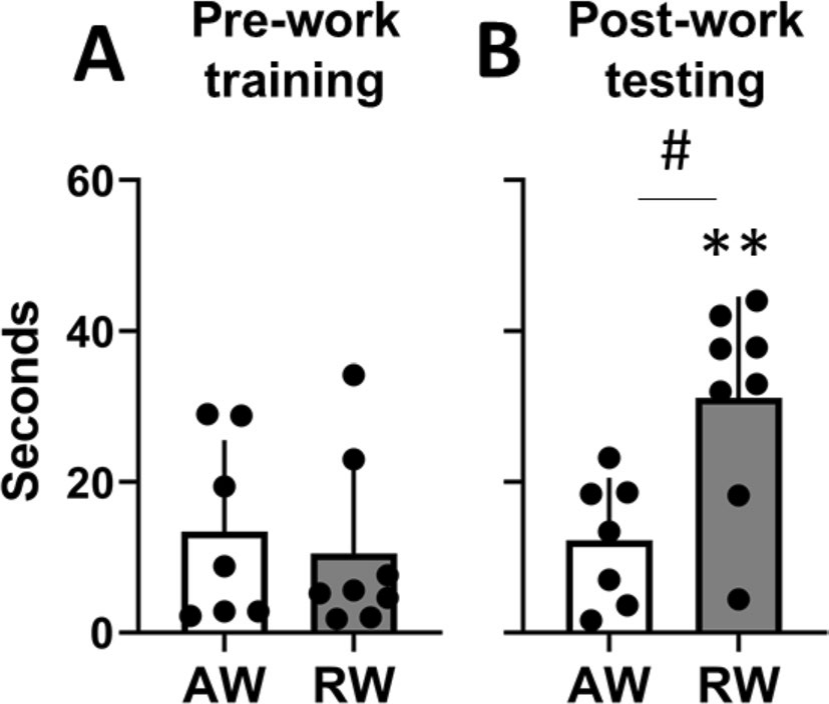
Latency to platform on the Morris Water Maze task, for active workers (AW) and rest workers (RW). (**A**) The last training trial before commencing the 3-day work period. Training occurred around zeitgeber time, ZT6. (**B**) The first testing trial immediately after the third work shift, AW at ZT20, RW at ZT10. Plots show mean ± SEM, with scatter plot overlaid. N = 7–8/group. **p < 0.01, compared to pre-work training. ^#^p < 0.05, between groups.

At the end of the 3-day training period, before the 3-day work period, all animals were able to locate the hidden platform in the Morris Water Maze (MWM) task in less than 40 s, with no significant difference between groups (t_(13)_ = 0.47, p = 0.65, d = 0.24; Fig. 2A). When tested immediately after the third work shift, rest workers required longer time to find the hidden platform compared to their pre-work training session (t_(7)_ = 5.04, p = 0.002, d = 1.64), and compared to active workers (t_(13)_ = 2.81, p = 0.014, d = − 1.40; Fig. 2B). Active workers’ latency post-work did not significantly differ from that during pre-work training (t_(6)_ = 0.21, p = 0.85, d = − 0.11). Latency to find the platform during pre-work training did not correlate with latency during post-work testing (r = 0.18, p = 0.52, all animals), indicating that post-work performance was independent of pre-work performance on the MWM task. Also, the differences in MWM performance cannot be explained by motor deficits following rest work as the swimming speed was higher in rest workers compared to active workers (mean 23.1 vs 16.0 cm/s; t_(13)_ = 2.56, p = 0.02, d = 1.33). Again, we observed considerable individual differences in spatial memory performance after simulated shift work (Fig. 2B).

### Type of work, markers of daily rhythm dynamics and sleep drive predict spatial memory performance

Having observed group differences, as well as considerable individual variation in performance on the MWM task, we decided to test whether this variation might be related to specific aspects of work, sleep and daily rhythmicity during the 3-day work period. To achieve this, we conducted a multiple regression analysis with hierarchical regression approach. This method allows to test whether certain variables can predict part of the variation in a second variable (as indicated by the R^2^ value), and whether adding more variables can enhance this prediction^22^. Adding variables to a model always enhances the R^2^. Therefore, we report the adjusted R^2^ which indicates predictive power after taking the addition of variables into account. If the adjusted R^2^ is increased after adding a given variable, this indicates that the variable indeed improves prediction.

First, we tested a simple model (Step 1, Table 1) with work condition as the only predictor of latency to platform in the MWM task. Work condition alone modestly predicted MWM performance (R^2^ = 0.23, Table 1). Next, we added circadian rhythm parameters (Step 2). Change in body temperature amplitude relative to baseline was chosen as a measure of global rhythm strength. REM sleep latency was chosen as a sleep-related marker of daily rhythmicity. This resulted in an increase in the adjusted R^2^ by 0.08 (Table 1), suggesting that individual changes to circadian rhythm parameters slightly enhances the ability of the model to predict MWM performance.

**Table 1:** Hierarchical regression analyses for predictors of Morris Water Maze (MWM) performance post simulated shift work.

	MWM latency to platform
**Step 1, work condition**	F_(1,11)_ = 3.96, p = 0.07
AW/RW	
R^2^	0.23
Adj. R^2^	0.20
**Step 2, daily rhythm dynamics**	F_(3,9)_ = 2.52, p = 0.12
Amplitude change	
REM sleep latency	
R^2^	0.46
Adj. R^2^ (change)	0.28 (0.08)
**Step 3, sleep drive**	F_(5,6)_ = 3.74, p = 0.07
Duration of NREM sleep bouts	
SWE in QW	
R^2^	0.76
Adj. R^2^ (change)	0.55 (0.27)

*AW* active work, *RW* rest work, *SWE* slow wave energy, *QW* quiet wakefulness, *REM* rapid eye movement sleep, *NREM* non-REM sleep.

Lastly, we tested whether adding sleep parameters further predicted MWM performance (Step 3). We applied cumulated slow-wave energy in quiet wakefulness and mean duration of the NREM sleep bouts across the last 24 h, since our previous findings suggest that these parameters are crucial markers of degraded waking function and sleep consolidation, and implicated in the observed effects of simulated shift work^17,21^. This resulted in a drastic increase in the adjusted R^2^ by 0.27, with the R^2^ reaching 0.76 (Table 1), suggesting that these sleep markers strongly contribute to predict individual spatial memory performance, following simulated shift work.

While cumulated slow-wave energy in quiet wakefulness contributed to predict MWM task performance, it is possible that this could reflect differences in NREM sleep intensity. To test this, we ran a second model where Step 3 was changed to cumulated slow-wave energy in NREM sleep (Table S1). This reduced the adjusted R^2^, suggesting that cumulated slow-wave energy in NREM sleep did not contribute to predict spatial performance in the MWM test in our animal model.

We also tested a third model where Step 3 was changed to cumulated time spent in NREM and REM sleep across the 3-day shift work protocol (Step 3, Table S2). Adding the amount of sleep spent across the shift work protocol resulted in a reduction in the adjusted R^2^, suggesting that sleep time during the 3-day shift work period does not contribute to predict MWM task performance.

### Rest work impairs BMAL1-driven protein translation in the prefrontal cortex

**Figure 3:**
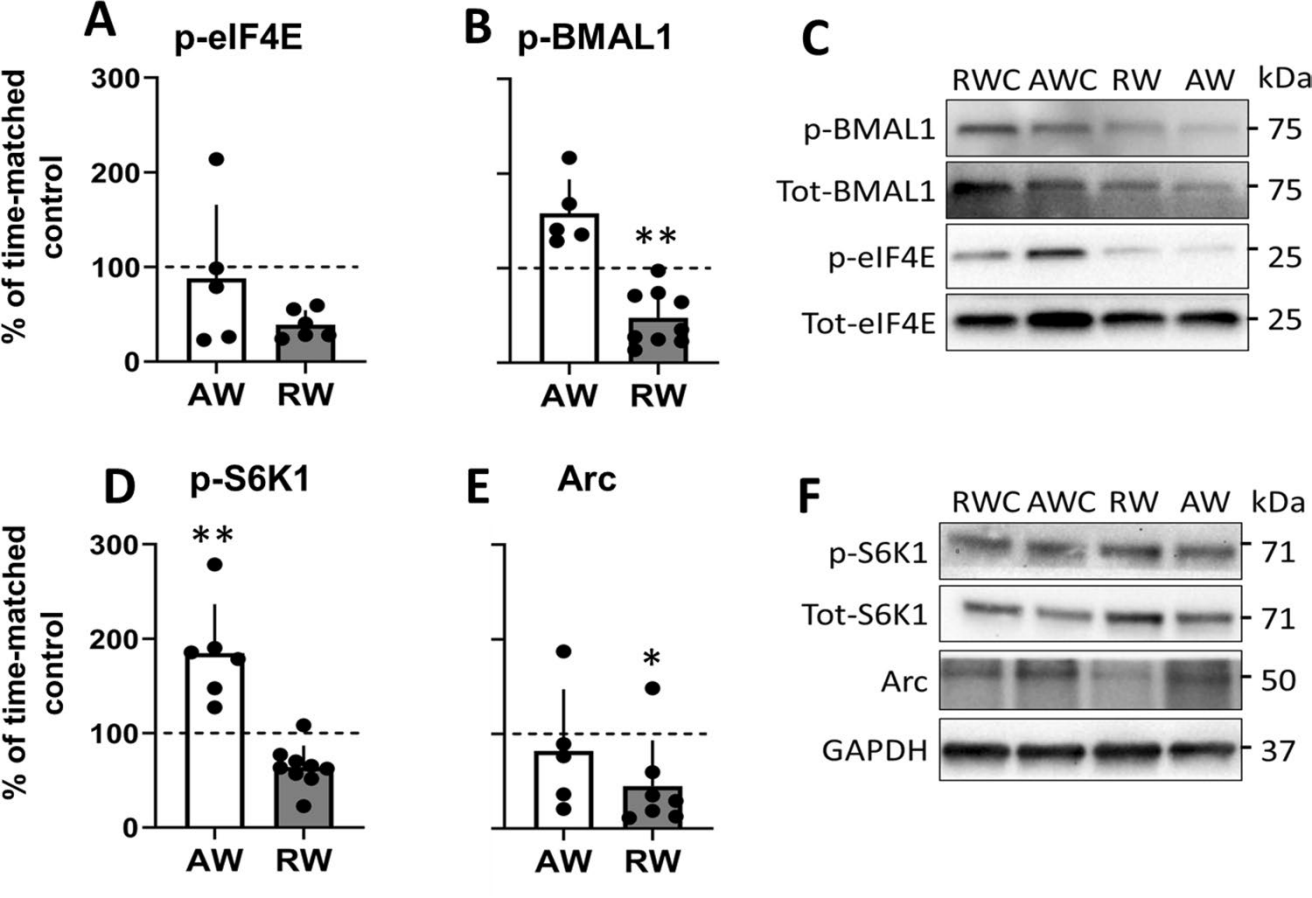
Expression of promoters of cap-dependent translation initiation, and synaptic plasticity regulators in prefrontal cortex following 3 days active work (AW, collected at zeitgeber time, ZT0) and rest work (RW, collected at ZT12). (**A**,**B**) m7GTP pull-down analysis. (**C**) Representative immunoblots for (**A**,**B**). Blots normalized to total eIF4E in the corresponding immunoblot. Phospho-protein normalized to total protein in the corresponding immunoblot. (**D**,**E**) Western blot analysis. (**F**) Representative immunoblots for (**D**,**E**). Full-length blots/gels are presented in Supplementary Figure S2. Blots normalized to GAPDH in the corresponding immunoblots. Phospho-protein normalized to total protein in the corresponding immunoblot. Quantification of immunoblots are expressed as percentage change relative to time-matched undisturbed control (AWC collected at ZT0, RWC collected at ZT12; normalized to 100%). Plots show mean ± SEM, with scatter plot overlaid. N = 6–9/group. *p < 0.05, **p < 0.01, compared to time-matched control.

The expression of the translational promoters, cap-bound phosphorylated BMAL1 (p-BMAL1) and p-eIF4E, was not significantly changed in the prefrontal cortex (PFC) of the active work rats compared to their time-matched undisturbed controls (p-BMAL1 t_(12)_ = 0.90, p = 0.39, d = 0.48; p-eIF4E t_(10)_ = 0.11, p = 0.92, d = 0.06; Fig. 3A–C). In contrast, expression of p-BMAL1 was significantly reduced in the rest workers compared to their time-matched controls (t_(13)_ = 3.19, p = 0.007, d = − 1.68; Fig. 3B), with large effect size for reduced p-eIF4E (t_(10)_ = 2.56, p = 0.078, d = − 1.48; Fig. 3A). The reduced phosphorylation of BMAL1 indicates repression of translation in rest workers since p-BMAL1 is coupled to stimulation of protein translation^16^.

Western blot analyses performed in the input samples (total tissue lysates used for cap-pulldown) showed no significant differences in protein expression, either from time-matched controls (indicating no time-of-day effect) (p-eIF4E AW t_(13)_ = 0.59, p = 0.57, d = 0.30; p-eIF4E RW t_(14)_ = 0.80, p = 0.44, d = 0.40; p-BMAL1 AW t_(12)_ = 0.74, p = 0.47, d = 0.40; p-BMAL1 RW t_(11)_ = 1.22, p = 0.25, d = 0.68; Fig. S1) or between the two work groups (indicating no effect of type of shift work) (p-eIF4E t_(13)_ = 1.1, p = 0.29, d = 0.55; p-BMAL1 t_(31)_ = 0.29, p = 0.78, d = 0.15; Fig. S1).

Levels of p-S6K1, the catalyst for BMAL1 phosphorylation, were significantly increased following active work (t_(11)_ = 3.21, p = 0.008, d = 1.79; Fig. 3D) compared to time-matched controls. Following rest work the p-S6K1 expression showed large effect size for reduced expression, although not statistically significantly different, compared to time-matched controls (t_(12)_ = 1.97, p = 0.07, d = − 1.10 Fig. 3D).

The expression of the synaptic plasticity regulator activity-regulated cytoskeleton-associated protein (Arc) was not significantly changed following active work (t_(11)_ = 0.27, p = 0.80, d = − 0.15; Fig. 3E,F), but it was significantly reduced following rest work (t_(12)_ = 2.21, p = 0.047, d = − 1.18; Fig. 3E), compared to their respective time-matched controls.

Taken together, these results replicate our previous findings that simulated night shift work impairs regulation of cap-dependent initiation of translation as well as expression of the synaptic plasticity regulator Arc^11^.

### Simulated shift work alters serum corticosterone

**Figure 4:**
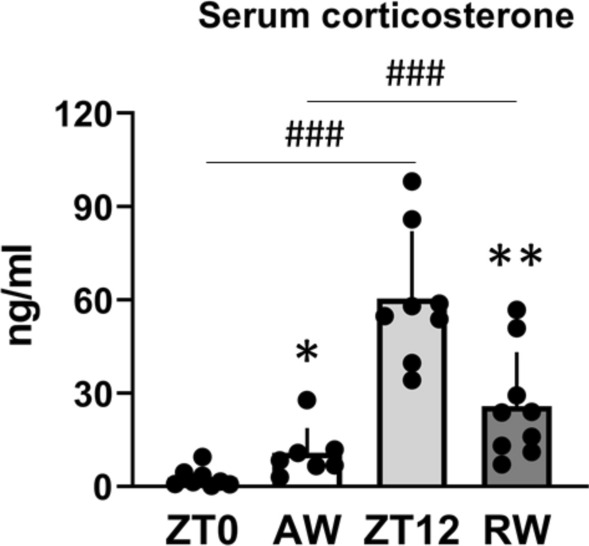
Concentration of serum corticosterone in undisturbed controls (zeitgeber time, ZT0 and ZT12) and following 3 days active work (AW, ZT0) and rest work (RW, ZT12). Plots show mean ± SEM, with scatter plot overlaid. N = 7–9/group. *p < 0.05, **p < 0.01, compared to time-matched controls. ^###^p < 0.001, between groups.

Serum corticosterone showed normal time-of-day variation in time-matched controls, with higher levels at ZT12 before the animals enter their active phase, compared to ZT0 before the animals enter their resting phase (t_(14)_ = 7.46, p < 0.001, d = 1.21; Fig. 4). Following active work, at ZT0, serum corticosterone was significantly increased relative to time-matched undisturbed controls (t_(13)_ = 2.61, p = 0.022, d = 1.31; Fig. 4). In contrast, serum corticosterone was significantly reduced at ZT12 in animals that had been subjected to rest work, relative to time-matched controls (t_(15)_ = 3.65, p = 0.002, d = − 1.76), but was higher compared to AW (t_(14)_ = 5.96, p < 0.001, d = 3.14; Fig. 4). These results suggest that simulated shift work alters the concentration of serum corticosterone, likely through changes in total concentration, diurnal profile, or both.

### Type of work, markers of daily rhythm dynamics and sleep drive and serum corticosterone predict different aspects of cap-dependent translation in the PFC

To evaluate whether work condition, parameters related to daily rhythmicity or sleep and level of stress hormone predict cortical expression of translational promoters and synaptic plasticity regulators, we conducted a multiple regression analysis with hierarchical regression approach, as described above.

Work condition strongly predicted expression of p-S6K1 (the p-BMAL1 regulator) (R^2^ = 0.87). All tested steps for p-S6K1 reached significance (p’s < 0.005; Table 2), but only very slight changes to the adjusted R^2^ across the steps show that this was due to the strong predictive effects of work condition on this protein. For all other examined proteins, work condition provided some predictive power (Step 1, Table 2).

**Table 2:** Hierarchical regression analyses for predictors of prefrontal cortex protein expression following simulated shift work.

	p-eIF4E	p-BMAL1	p-S6K1	Arc
**Step 1, work condition**	F_(1,7)_ = 3.98, p = 0.09	F_(1,11)_ = 8.68, p = 0.013	F_(1,11)_ = 72.39, p < 0.001	F_(1,8)_ = 1.76, p = 0.22
AW/RW				
R^2^	0.36	0.44	0.87	0.18
Adj. R^2^	0.27	0.39	0.86	0.08
**Step 2, daily rhythm dynamics**	F_(3,5)_ = 2.59, p = 0.17	F_(3,9)_ = 5.07, p = 0.03	F_(3,9)_ = 22.58, p < 0.001	F_(3,6)_ = 0.49, p = 0.70
Amplitude change				
REM sleep latency				
R^2^	0.61	0.63	0.88	0.20
Adj. R^2^ (change)	0.37 (0.10)	0.50 (0.11)	0.84 (− 0.02)	− 0.20 (− 0.28)
**Step 3, sleep drive**	F_(5,2)_ = 1.93, p = 0.38	F_(5,6)_ = 2.10, p = 0.20	F_(5,6)_ = 14.75, p = 0.003	F_(5,3)_ = 0.82, p = 0.61
Duration of NREM sleep bouts				
SWE in QW				
R^2^	0.83	0.64	0.92	0.58
Adj. R^2^ (change)	0.40 (0.03)	0.33 (− 0.17)	0.86 (0.02)	− 0.12 (0.08)
**Step 4, serum corticosterone**	F_(6,1)_ = 35.51, p = 0.13	F_(6,5)_ = 1.96, p = 0.24	F_(6,5)_ = 14.47, p = 0.005	F_(6,2)_ = 0.82, p = 0.64
R^2^	0.99	0.70	0.95	0.71
Adj. R^2^ (change)	0.97 (0.57)	0.34 (0.01)	0.88 (0.02)	− 0.16 (− 0.04)

*AW* active work, *RW* rest work, *SWE* slow wave energy, *QW* quiet wakefulness, *REM* rapid eye movement sleep, *NREM* non-REM sleep.

#### Daily rhythm parameters predict cap-binding activity of translational promoters

Adding daily rhythm parameters to our hierarchical regression model (Step 2) enhanced the predictive power of expression of the cap-bound translational promoters p-eIF4E and p-BMAL1 (by 0.10 and 0.11, respectively; Table 2).

We performed a separate regression analysis to test whether these parameters also predict expression of total available protein in total tissue lysates. In the case of p-eIF4E, rhythm parameters also predicted expression in total tissue lysates (increased adj. R^2^ by 0.16, Table S3), suggesting an overall impact of rhythms on p-eIF4E abundance. In the case of p-BMAL1, rhythm parameters did not predict expression in total tissue lysates (reduced adj. R^2^ by 0.13, Table S3), suggesting that these specifically predict the cap-binding activity of p-BMAL1.

#### Sleep drive during the last 2 h marginally predicts the expression of translational promoters and the regulator p-S6K1

To examine the contribution of acute changes in sleep drive on translational promoters, we tested a model including these sleep parameters, as above (Step 3). Since protein expression and cap-binding activity are thought to reflect relatively short-term (minute- and hour-scale) biological processes, we used data starting after the third work shift when rats were returned to their home cage, until tissue collection 2 h later. Results show that markers of sleep drive during this period slightly predict the expression of cap-bound p-eIF4E (adj. R^2^ increased by 0.03, Table 2), as well as p-S6K1 (adj. R^2^ increased by 0.02, Table 2). These markers also predicted phosphorylation of eIF4E in total tissue lysates (adj. R^2^ increased by 0.36, Table S3).

Since markers of sleep drive during wakefulness and sleep consolidation predicted protein expression, we tested a separate model to investigate whether this was also the case for accumulation of slow wave energy during NREM sleep (as a marker of sleep intensity). Slow-wave energy accumulation during NREM sleep across the 2 h time-window reduced the adjusted R^2^ or resulted in negative adjusted R^2^ across all proteins tested (Step 3, Table S4). Thus, slow-wave energy in NREM sleep did not contribute to predict any of the measured proteins.

Testing a separate step with markers of cumulated total sleep time (NREM sleep and REM sleep respectively) across the 3-day work period only slightly enhanced prediction of cap-bound p-BMAL1 (adj. R^2^ increased by 0.01, Step 3, Table S5), with no enhanced prediction of any other measured proteins (Table S5).

Taken together these results suggest that the last 2-h measures of sleep drive to a slight extent, but not NREM sleep intensity or cumulated sleep across the work protocol, contribute to prediction of eIF4E and S6K1 phosphorylation in PFC after simulated shift work.

#### Serum corticosterone levels predict expression of the translational promoter p-eIF4E

To assess the contribution of serum corticosterone to the prediction of cortical protein activity, we added Step 4 (concentration of serum corticosterone) to our hierarchical regression model. Serum corticosterone drastically elevated the prediction of the translational promoter cap-bound p-eIF4E (adjusted R^2^ increased by 0.57, Table 2). Serum corticosterone also enhanced the prediction of p-eIF4E expression in total tissue lysates (adjusted R^2^ increased by 0.13, Table S3), suggesting that concentration of corticosterone may predict abundance of p-eIF4E in the PFC following simulated shift work. Serum corticosterone only slightly enhanced the prediction of cap-bound p-BMAL1 (adjusted R^2^ increased by 0.01, Table 2) and p-S6K1 (adjusted R^2^ increased by 0.02, Table 2).

The expression of the synaptic plasticity regulator Arc was only predicted by work condition, not by any of the selected markers of circadian rhythmicity, sleep drive or serum corticosterone (negative adj. R^2^ suggesting no fit of any of the models, Table 2).

## Discussion

The aim of the present study was to identify potential mechanisms underlying the cognitive deficits associated with shift work. We utilized a rat model where animals were exposed to either simulated night shift work (rest work) or simulated day shift work (active work) for three consecutive days. Results show that individual variation in daily rhythm dynamics, short-term markers of sleep drive and serum corticosterone predict individual variations in spatial memory impairments and the cerebral cortical protein translational machinery after simulated shift work. These findings open new research avenues into the biological mechanisms underlying individual variation in shift work tolerance.

### Effects of simulated shift work at the group level

At the group level, we observed effects of rest work on sleep and wakefulness as previously observed in our model. While simulated night shift work impacts on the temporal distribution of sleep throughout the 24-h day in our model, it does not affect overall time spent in sleep differently from simulated day shift work^17^. Moreover, slow-wave energy accumulation during NREM sleep did not differ between the groups. Nevertheless, rest work is associated with a degraded waking state, as evidenced by increased slow wave energy in quiet wakefulness during the simulated night shift period^17^. In the present study, we replicate this finding. This may be explained by global instability in arousal including OFF periods of neuronal firing typically associated with a local slow wave (which causes intermittent performance impairments)^23^ or incomplete dissipation of homeostatic sleep drive^24^ after simulated night shift work. Our previous data showed a lower build-up of sleep debt in the rest workers than in active workers, probably due to the slowing of EEG during quiet wakefulness^17^. A lower build-up of sleep drive in rest workers after work-hours may very well be explained by the time spent in sleep before work together with a recent finding in mice that quiet wakefulness is permissive to metabolic changes that occur in NREM sleep^25^. Human studies have reported relatively well-preserved deep sleep (stage N3 sleep) after the night shift^26^. Taken together these data suggest that NREM sleep quality and quantity per se do not impact on brain functioning after the night shift, but that other aspects of sleep regulation may play an important part. In the present study we also show that cognitive performance is impaired after rest work, as evidenced by reduced spatial memory performance on the Morris Water Maze task. This indicates that our model mimics the cognitive performance impairments associated with human night shift work^1,27^. We also show that simulated shift work impacts on concentrations of serum corticosterone, as evidenced by a reduction in corticosterone 2 h after rest work, also observed in several human studies^28–30^.

Night shift work is associated with impairments to the circadian rhythm, in particular to what has been termed internal desynchronization^31,32^. In our study we find overall group differences in two separate bodily rhythms following rest work,the body temperature rhythm amplitude and REM sleep latency. Taken together, these results show that our model captures several aspects associated with human shift work.

### Individual differences in tolerance to shift work

In most of the measured outcomes during and following simulated shift work, we observed considerable individual variation, with overlap between active and rest workers. Some of the measures in our observed group effects are undoubtedly influenced by the time-of-day in which animals return home from work and data is collected, as is also the case for humans that work shifts. Nevertheless, the individual differences indicate variation in shift work tolerance in our rat model, as has been documented in several human studies^18,19^. Changes to circadian rhythm, sleep and stress systems have been hypothesized as mechanisms contributing to the negative effects of shift work. Considering this, we tested whether changes to related parameters during the 3-day work protocol might predict the observed effects on cognitive performance and cortical protein expression in our model.

### Markers of daily rhythm dynamics and sleep drive predict cognitive performance after simulated shift work

Markers of daily rhythmicity, and more strongly, markers of sleep drive in the last 24 h, predicted individual differences in spatial memory performance after simulated shift work. In human shift workers, sleep has to date been most clearly implicated as a predictor of cognition in general^3,4^ and cognitive deficits in shift work specifically^5^. Our results suggest that changes in sleep drive during and between shifts are of importance, rather than sleep quality or cumulated time spent in sleep across the shift work protocol per se.

While sleep is important for maintaining cognitive performance^33^, so is circadian rhythmicity^34^. The present model of night shift work has been shown to shift feeding rhythms toward the rest phase, with acute negative effects on markers of metabolic functioning^35^. Another recent study showed that similar manipulation of feeding rhythms also impaired cognitive performance and hippocampal functioning^36^. However, a different study failed to find any effects of time-restricted feeding on cognitive performance, despite multiple attempts^37^. Nevertheless, these findings highlight that circadian rhythm disturbance can reflect simultaneous impairments to multiple systems. Very few studies examine the interaction between circadian rhythms and sleep on cognitive performance, although some biochemical pathways implicated in memory performance and consolidation have been shown to be altered by both sleep and circadian rhythms. These for example include the cAMP/MAPK/CREB transcriptional pathway^38^, but more recently also the cap-dependent translation initiation pathway^11,16,39,40^.

### Daily rhythm, sleep drive and serum corticosterone predict markers of cortical regulation of translation after simulated shift work

We have previously suggested that impairments to eIF4F complex assembly after rest work may in part underlie the cognitive deficits observed on the night shift^11^. The present study strengthens this notion. We replicate previous findings from our lab showing that the regulation of cap-bound translational regulators (eIF4E and BMAL1) is impaired in the prefrontal cortex following rest work, as compared to undisturbed time-matched controls^11^.

We show that expression of the translational regulators p-BMAL1 and p-eIF4E within the PFC are predicted by changes in daily rhythmicity during simulated shift work. Phosphorylated BMAL1 was predicted by daily rhythmicity only in its mRNA-cap-bound form. While it was previously known that BMAL1 acts both as a clock protein and as a translational enhancer when phosphorylated^16^, it was not clear whether its cap-binding activity is in fact affected by altered rhythms. The present results support the idea that p-BMAL1 translational activity is indeed regulated, or at least modulated, by overall changes to daily rhythmicity during simulated shift work.

Our data also point to a role for daily rhythmicity in the modulation of the translational regulator eIF4E. Phosphorylation state of eIF4E was predicted by parameters of daily rhythmicity, acute (2 h) markers of sleep drive and serum corticosterone. Few studies examine the interaction of circadian rhythms, sleep and/or corticosterone on eIF4E expression or on any related marker. However, eIF4E and several other proteins related to cap-dependent translation have been shown to oscillate in a circadian fashion in different brain regions^16,41,42^. A study by Tudor and colleagues showed that biochemical enhancement of cap-dependent translation by indirect promotion of eIF4E phosphorylation rescued sleep deprivation-associated cognitive deficits on the MWM task^40^, providing a link between cap-dependent translation, sleep and cognitive performance.

We also observed individual changes in concentration of serum corticosterone after shift work, which predicted expression of several translational markers in the PFC, particularly eIF4E phosphorylation. While corticosterone is commonly referred to as a stress hormone, the observed changes in serum corticosterone cannot be said to reflect acute stress, considering that the levels were measured 2 h after ended simulated shift work. Two hours after the end of sleep restriction, or the onset of restraint stress, it has been shown that corticosterone levels are returned basal levels^43,44^. However, it is possible that changes may reflect longer-term alterations to the regulation of the HPA-axis-mediated stress system, as has been observed after human shift work^8–10^ and in a rat model of long-term simulated shift work^45^. One study by Grønli and colleagues showed that p-eIF4E expression in the PFC was associated with prior sleep time and quality, but this association was abolished after chronic stress, highlighting a potential interaction between stress and sleep on translational regulation^39^. The PFC is known to be sensitive to stress in general and corticosteroids specifically^46^, and stress and sleep interact to impact on synaptic plasticity and cognitive functioning in a complex way^47^. More studies are needed to further elucidate how circadian rhythm, sleep and stress systems together may affect cap-dependent translational processes specifically, and cognitive functioning more generally.

Our results showed that the BMAL1-regulator S6 Kinase 1 (S6K1) was strongly predicted by work condition, and slightly predicted by markers of sleepiness and sleep consolidation. A recent study showed that several protein kinases oscillate in abundance within forebrain synapses, mostly peaking at light–dark transitions^48^. Interestingly, sleep deprivation resulted in the loss of rhythmic expression of almost all previously identified rhythmic kinases, highlighting the sensitivity of kinases to sleep deprivation^48^. Another recent study showed that 10 h acute sleep deprivation impaired striatal S6K1 signalling, and performance on a motor task^49^. Inhibition of S6K1 also impaired motor performance, suggesting a link between S6K1 signalling, sleep and brain functioning^49^. We have previously shown that p-S6K1 expression varies significantly between time points of light–dark transitions (ZT0 vs ZT12) in the PFC of undisturbed animals, and that the high expression of p-S6K1 at ZT12 is abolished after rest work^11^. Considering previous literature and the present data, this outcome may be primarily related to effects of sleep deprivation on kinase stability.

Acting through the corticosteroid receptor, circulating corticosterone is likely to have a significant impact on S6K1 function as S6K1 phosphorylation is subject to inhibition by the corticosteroid receptor^50^. The apparent inversion of this relationship (low corticosterone coupled with reduced p- S6K1 in rest workers relative to active workers) is paradoxical. Yet the reductions in corticosterone concentration and p-S6K1 could both be explained as reactions to elevated glucocorticoid receptor stimulation, not at the time of euthanasia, but hours before, for instance early in the rest phase work session. A longitudinal profile of serum corticosterone from throughout the work sessions would be needed to address this possibility.

Arc protein is recognized as a critical regulator of activity-dependent synaptic plasticity in the mammalian brain^51^. The expression of Arc in individual animals was not predicted by any of the examined processes,circadian rhythmicity, sleep drive, total cumulated sleep time, NREM sleep intensity or serum corticosterone. Previous studies associate Arc protein expression with total sleep time^47^. In our case, both groups were exposed to 8 h forced activity, followed by 2 h home cage recovery prior to tissue collection. Thus, there may not have been sufficient individual variation in cumulated sleep time to identify differences in Arc expression between animals in our study.

### Study limitations

Some limitations of the present study warrant discussion. Firstly, cognitive testing as well as assessment of protein synthesis and serum corticosterone levels were performed at one time point per group (rest workers at ZT10/ZT12, active workers at ZT22/ZT0). It cannot be excluded that part of the differences observed post-work can be attributed to time-of-day differences in time of testing. However, several studies show no time-of-day effects on performance on the MWM and related spatial tasks in nocturnal rodents^34,52–55^. Moreover, rest workers were trained and tested during their resting phase, at time-points closer to each other (ZT6 and ZT12 respectively), as opposed to active workers (ZT6 and ZT22 respectively). Thus, if recall were context-dependent one might expect RW to perform better on the task, while they in fact performed much worse post-work both compared to their pre-work training sessions and to the active workers. When it comes to serum corticosterone, we cannot conclude whether changes are due to effects on total concentration or diurnal profile. REM sleep latency was selected as a sleep-dependent daily rhythm marker, and between-group differences were likely influenced by work at different circadian phases^20,56^. However, of importance to our study was the individual variation in cognitive performance, serum corticosterone concentrations and REM sleep latency, which overlapped between groups.

Secondly, hierarchical regression is inherently subjective, as predictors are chosen based on previous literature and hypothesized associations. However, the approach allows the inclusion of several predictors at multiple levels, and does not require large data sets, as with related machine-learning-based approaches. Our approach proved capable of identifying large effect sizes, indicating sufficient sensitivity even with a small number of animals. While the analysis can be criticised as underpowered, it raises several new hypotheses which can be tested with appropriate study designs. Future research could aim to manipulate markers of daily rhythm strength, sleep drive and/or serum corticosterone and measure the effect on MWM performance and PFC protein expression. For example, markers of sleep drive could be manipulated with environmental (e.g. light) or pharmacological agents. Such interventions would thus be hypothesized to directly impact on MWM performance and PFC protein expression, suggesting a causal link between these measures.

Thirdly, it would be of interest to inform whether the observed individual variability in shift work tolerance reflect trait or stochastic differences. However, the present experimental design does not allow us to assess this. One might consider comparing individual variation in shift work tolerance across days, but this could potentially reflect adaptations or additive effects of the simulated work protocol. Therefore, this issue should be addressed in future work with adapted study designs.

Lastly, we only included male rats in the present study. Human studies suggest that gender may modulate shift work tolerance, with women being more vulnerable to the short-term effects of shift work on sleep and cognitive performance, and men being more vulnerable to the longer-term health consequences^18^. While social and cultural aspects are likely important here, animal studies may aid in elucidating possible biological mechanisms underlying this potential dissociation.

## Conclusion

In summary, we have demonstrated that in our rat model, simulated night shift work induces detrimental effects on cognitive performance and markers of brain plasticity, with considerable individual variation in shift work tolerance. Additionally, we show that markers of daily rhythmicity, sleep drive and serum corticosterone all predict various aspects of these outcomes. Notably, total sleep time did not predict any outcomes of cognitive performance and brain plasticity. Future studies should further investigate how these processes may interact to impair cognitive performance and brain functioning, both in shift workers and in other populations.

## Methods

### Ethics

This study was conducted in accordance with Norwegian laws and regulations, and The European Convention for the Protection of Vertebrate Animals used for Experimental and Other Scientific Purposes. The protocol was approved by the Norwegian Food Safety Authority (permit number: 11321).

### Animals and housing

Male rats of the outbred Sprague-Dawley strain (n = 32, 16 experimental animals and 16 controls, nTac:SD; Taconic, Silkeborg, Denmark) were used in the study. At arrival the animals were weighing approximately 300 g. After acclimatization to the laboratory conditions, they were group housed in individually ventilated cages (IVC, Techniplast, 75 air changes/h) type IV (480 × 375 × 210 mm, 1,500 cm^2^) and single housed during the experimental protocol (IVC cage type III, 425 × 266 × 185 mm, 800 cm^2^). The animals were kept on a 12 h light/12 h dark schedule with lights on at 08:00 (zeitgeber time 0; ZT0). Mean light intensity during lights ON was 222 ± 112 lx. Food (Rat and mouse No. 1, Special Diets Services) and filtered water were available ad libitum throughout the experiment.

### Surgery

Experimental animals were implanted with transmitters (Physiotel, Data Sciences International) for continuous wireless recording of body temperature and sleep/wake electroencephalography (EEG) and electromyography (EMG), as previously described^57^. In brief, animals were anaesthetized with subcutaneous injection of a mixture of fentanyl 0.277 mg/kg, fluanisone 8.8 mg/kg (Hypnorm, Janssen) and midazolam 2.5 mg/kg (Midazolam, Actavis), and the transmitters were placed in subcutaneous pockets in the dorsomedial lumbar region (4ET transmitters) or in the neck region (F40-EET transmitters). Intracranial electrodes for collection of EEG signals were secured to the skull with dental acrylic (GC Reline, America inc.); frontal-frontal derivation bregma coordinates: AP = 2.0 mm, ML =  − 2.0 mm and lambda coordinates: AP = 2.0 mm, ML = 2.0 mm; and frontal-parietal derivation bregma coordinates: AP = 2.0 mm, ML =  − 2.0 mm and lambda coordinates: AP = 2.0 mm, ML = 2.0 mm). Two electrodes were attached to the neck muscle for collection of EMG signals. Animals were allowed 14 days recovery before entering the experiment^58^.

### Overview of study design

The study was conducted in two parts; (1) to test the effect of simulated shift work on spatial memory performance; (2) to collect tissue for protein analysis following simulated shift work. Telemetric recordings were conducted throughout the experimental period.

In experiment 1, all animals underwent 5 days of baseline telemetric recordings, followed by 3 days of training on the Morris Water Maze (MWM) task. They were then exposed to 3 consecutive days of simulated shift work, during which they were randomized to either simulated night shift work (rest work; RW) or simulated day shift work (active work; AW). Immediately after the third shift, recall on the MWM task was tested.

Animals were allowed at least 4 weeks washout between experiments. In experiment 2, animals were again recorded for 5 days baseline and randomized to either rest work or active work for 3 consecutive days (yielding n = 16 data points per work condition for sleep and circadian parameters). Two hours after the third shift, animals were euthanized for blood and brain collection^11^. A separate batch of animals (n = 16; undisturbed controls), were single housed for 14 days before tissue collection.

### Simulated shift work procedure

To simulate shift work, animals were exposed to forced activity for 8 h per day, centred either in their normal active phase (active work; ZT14–22) or in their normal rest phase (rest work; ZT2–10), as described previously^11,17,35^. Animals were placed in automatically rotating wheels (Rat Running Wheel, TSE Systems, Homburg, Germany,24 cm diameter; 3 rpm; 1,440 revolutions or approx. 1.1 km of linear distance per 8-h session). Food and water were available ad libitum. Rotating wheels, feeders and water bottles were cleaned after each work session with 5% ethanol solution. Between sessions, animals were housed in their home cage. Work schedules were repeated for 3 consecutive days.

### Morris Water Maze task

For assessment of spatial memory performance, animals were subjected to the Morris Water Maze (MWM) task^59^. Training and testing occurred in a circular pool (diameter 130 cm, height 60 cm) filled with water (maintained at 25 ± 1 °C). Water was re-filled for each day of testing. On the walls inside and around the pool were spatial cues (coloured symbols) for navigation.

Prior to shift work, all animals received 3 days of training. Each animal had four trials per day starting around ZT6. All training and testing were performed by the same experimenter under dim red light conditions. Animals were released from one of eight starting positions and had to swim to reach a platform (11 cm^2^, transparent plexiglass) on which they were allowed 15 s of rest before commencing the next trial. Trials were organized in semi-randomized order; the animals were never released from the same starting position twice in a row. On the first day, the platform was located in the middle of the pool, visible above the water surface. On the next two days, the platform was located in one specific quadrant of the pool, hidden below the water surface. Black paint (Tempera) was added to the water to make it opaque to prevent the rats from seeing the platform. If the animal failed to find the hidden platform within 120 s, it was guided to the platform by the experimenter, and a score of 120 s was given. One animal was unable to learn the platform location and consequently excluded from further analysis.

Immediately after the third work shift, the animals’ recall of the platform location was tested (rest workers at ZT10, active workers at ZT22). EthoVision XT software (Noldus) was used for the acquisition and processing of data. Latency to platform and swim speed was calculated for each animal.

### Telemetric recordings and processing of data

The wireless recording device (input voltage: − 1.25 to + 1.25 mV) acquired EEG and EMG signals at a sampling rate of 250 Hz. Body temperature was sampled at 50 Hz and data was stored per 10 s (Dataquest A.R.T, version 4.1, Data Sciences International). Telemetry signals were collected through receivers (RPC-2/RPC-3, Data Sciences International) placed directly beneath the animals’ home cage or next to the rotating wheel during simulated shift work.

#### Processing and analysis of EEG and EMG data

Based on the EEG and EMG signals and in accordance with the criteria from Neckelmann and Ursin^60^, wakefulness, non-REM (NREM) sleep and REM sleep was manually scored in 10 s epochs using Neuroscore software (version 3.2.0, Data Sciences International). For the purpose of scoring, EEG signals were filtered with high-pass at 0.5 Hz and low-pass at 35 Hz. EMG signals were filtered with high-pass at 5 Hz. We further distinguished quiet wakefulness from active wakefulness as those epochs of wakefulness in which the EMG peak-to-peak amplitude was ≤ 33rd percentile of all wakefulness EMG peak-to-peak amplitude values^25^.

We calculated the average NREM sleep bout length, as previous mathematical modelling of data suggests that differences in NREM sleep bout duration importantly contribute to the observed effects of simulated shift work on sleep/wake dynamics in our model^21^. A NREM sleep bout was defined as 3 or more consecutive 10-s epochs of NREM sleep.

REM sleep latency was calculated as a sleep-related marker of circadian rhythmicity, as similar to humans REM sleep specifically has been shown to be regulated by the circadian system in rats^20^. This was defined as the latency to 3 consecutive 10-s epochs of REM sleep, upon return to the home cage after a work session^17^. Latency was calculated after the second work session since not all animals entered REM sleep after the third session (before tissue collection 2 h later). Similarly, latency to stable sleep was defined as latency to 6 consecutive 10-s epochs of sleep, upon return to the home cage after the second work session.

In addition to time spent in different vigilance states, we also analysed the spectral characteristics of the EEG during these states, using offline Fast Fourier Transform analysis on unfiltered EEG signals averaged across consecutive 10-s epochs. The procedure has been described in detail elsewhere^61^. Artefacts were removed by visual inspection of EEG signals, and an automated algorithm that detected epochs with EEG power spectral values that exceeded the mean value by at least 5 standard deviations. EEG spectral power tracked across states was normalized to the average power values of these vigilance states at the corresponding circadian time during baseline of each animal.

We calculated EEG slow-wave activity (SWA, average spectral power in the 1–4 Hz range) in the fronto-parietal EEG derivation in NREM sleep as a measure of sleep intensity, and in quiet wakefulness as a measure of the homeostatic sleep drive^61^. The accumulation of SWA over time (slow-wave energy, SWE) in quiet wakefulness and NREM sleep was calculated by multiplying the number of epochs in the specific state per 2 h with the average SWA in the given state for those hours.

#### Processing and analysis of body temperature data

Processing and analysis of body temperature data was performed using the *tidyverse* package in R software^62,63^. For each animal, body temperature was normalized to baseline (4-day baseline average set to 0). Artefacts were removed by a moving window over 100 data points at a time, excluding all values 2 °C above or below the mean, and 5 standard deviations above or below the mean. The 10-s temperature measurements were averaged to 5-min bins. The amplitude of the body temperature rhythm was applied as a measure of change in overall rhythm strength during work. This was analysed with cosinor analysis, using the *cosinor* package in R software^64^. Analysis was performed on 72 h baseline data and 72 h work data. Rhythm amplitude change from baseline was calculated by subtraction.

### Tissue collection

After the third simulated work session, animals were returned to their home cage for 2 h before they were anesthetized with isoflurane, and euthanized by decapitation (AW at ZT0 and RW at ZT12). Undisturbed animals never exposed to work sessions were used as time-matched controls and sacrificed at ZT0 (n = 8) and ZT12 (n = 8).

Trunk blood was collected in serum tubes, left to coagulate on ice in serum tubes for 30 min, and subsequently centrifuged at room temperature (3,000 rpm for 10 min). Serum was aliquoted and stored at − 80 °C until analysis. The time from initial disturbance of the animal (by removing the cage from the rack) to complete blood collection was 2:15 ± 0:18 min.

Brains were quickly removed and bilateral PFC was dissected out since previous research from our lab showed biochemical alterations in PFC after shift work^11^. The tissue was flash frozen and stored at − 80 °C until analysis.

### M7GTP (cap) pull-down and SDS-PAGE procedure

m7GTP (cap) pull-down assay was conducted in order to assess changes in the abundance and cap-associated total eIF4E, Ser209 phosphorylated eIF4E, total BMAL and Ser42 phosphorylated BMAL^65^. Bilateral PFC was homogenized in 1,000 µl of RIPA buffer (Pierce, Thermo Scientific) with added protease and phosphatase inhibitor (Halt, Thermo Scientific), and centrifuged for 10 min at 14,000g at 4 °C. For the cap pull-down assay, 400 µg of protein in addition to 30 µl of 7-methyl GTP-agarose beads (Jena bioscience #AC-141) were incubated for 4 h at 4 °C. Phosphate-buffered saline was used to wash the beads three times, then boiled in Laemmli sample buffer (Bio-Rad) and sample was resolved in SDS/PAGE gels (4–15% precast gels, Criterion TGX, Bio-Rad). Proteins were transferred to nitrocellulose membranes (Trans-Blot turbo transfer packs, Bio-Rad) which were then blocked with 5% non-fat dry milk, probed with antibodies and developed using chemiluminescence reagents (Pierce, #32106).

Antibodies used for immunoblotting were as follows: p-eIF4E (1:1,000, Cell Signaling #9741), eIF4E (1:1,000, Cell Signaling #9742), p-BMAL1 (1:1,000, Cell Signaling #13936), total BMAL1 (1:500; Santa Cruz Biotechnology #sc365645), p-pS6k (1:1,000, Santa Cruz Biotechnology #sc-7984), pS6k (1:1,000, Sigma #SAB4502691), Arc (1:500; Santa Cruz Biotechnology #sc17839), and GAPDH (1:5,000, Santa Cruz Biotechnology #sc32233).

Blots were scanned using Gel DOC XRS+ (Bio-Rad) and densitometric analyses were performed with ImageJ software (NIH). Densitometric values are expressed per unit of tot-eIF4E/GAPDH as specified, applied to the gel lane. Blots treated with phospho-specific antibody (p-eIF4E, p-BMAL1, p-pS6k) were stripped with stripping buffer (Restore western blot, Thermo Scientific) at 37 °C for 10 min, washed, blocked and re-probed with antibody recognizing total protein.

For this analysis the amount of cap-bound p-BMAL1 and total-BMAL1, was normalized to the total amount of cap-bound eIF4E. Phosphoproteins were normalized relative to the total protein on the same lane. Total proteins were normalized to loading control. Protein expression after simulated shift work was normalized to expression from time-matched controls.

### Serum corticosterone analysis

Serum corticosterone was analysed in triplicate with the ELISA technique (Enzo ADI-900-097, Enzo Life Sciences) according to the manufacturers’ instructions. The kit was selected based on previous data from our lab showing high correlation to other kits in addition to high detection of corticosterone even at low concentrations^66^. Values were averaged across the triplicates for each sample. The coefficient of variation (CV%) was accepted at < 20.

### Statistical analyses

Statistical analyses were performed using R software (version 3.6.1)^63^ and GraphPad Prism (version 8.2.1). Group differences in time spent in vigilance states was calculated using student’s unpaired t-test. Within-group differences in vigilance states on work-days vs baseline was calculated using student’s paired t-test. Group differences in latency to platform on the MWM task were computed using student’s unpaired t-test. Correlation between pre- and post-work performance on the MWM task was calculated using Pearson’s product moment correlation. Differences in serum corticosterone and PFC protein expression were compared between groups and relative to time-matched controls using Student’s unpaired t-test. Difference in daily rhythm parameters following work relative to baseline was computed using Student’s paired t-test. The alpha-value for significance was set to 0.05.

Cohen’s d was calculated as measure of effect size. For interpreting d, 0.2 is considered small, 0.5 medium and 0.8 large effect size^67^.

Multiple regression analysis using the hierarchical regression approach was performed to determine predictors of MWM task performance and PFC protein expression^22^. Included predictor variables were (1) work condition, (2) daily rhythm dynamics, including body temperature rhythm amplitude change and REM sleep latency, (3) sleep parameters, including (3a) average length of NREM sleep bouts and slow-wave energy in quiet wakefulness, (3b) total cumulated time spent in NREM sleep and REM sleep, or (3c) slow-wave energy in NREM sleep, and (4) serum corticosterone.

## Supplementary information

Supplementary Information.

## Data Availability

The datasets generated and analysed during the current study are available from the corresponding author on reasonable request.
