# Supplementary material for: Cognitive function and brain plasticity in a rat model of shift work: role of daily rhythms, sleep and glucocorticoids
Source: Sci Rep. 2020 Aug 4;10:13141. doi: 10.1038/s41598-020-69969-x (PMC7403587; doi:10.1038/s41598-020-69969-x)
Supplement: Supplementary file 1 — Supplementary Information. [file 41598_2020_69969_MOESM1_ESM.pdf]

Supplementary file

Article title: Cognitive function and brain plasticity in a rat model of shift work: role of daily rhythms, sleep and glucocorticoids

Authors: Andrea R Marti[1], Torhild T Pedersen[1], Jonathan P. Wisor[2], Jelena Mrdalj[1], Øystein Holmelid[1], Sudarshan Patil[3], Peter Meerlo[4], Clive R Bramham[3], Janne Grønli[1]

1 Bergen Stress and Sleep Group, Department of Biological and Medical Psychology, Faculty of Psychology, University of Bergen, Bergen, Norway

2 College of Medicine, Washington State University, Spokane, Washington, USA

3 Department of Biomedicine, Faculty of Medicine, University of Bergen, Bergen, Norway

4 Groningen Institute for Evolutionary Life Sciences, University of Groningen, Groningen, Netherlands

Corresponding author's address and current e-mail: Jonas Liesvei 91, 5009 Bergen, Norway, andrea.marti@uib.no

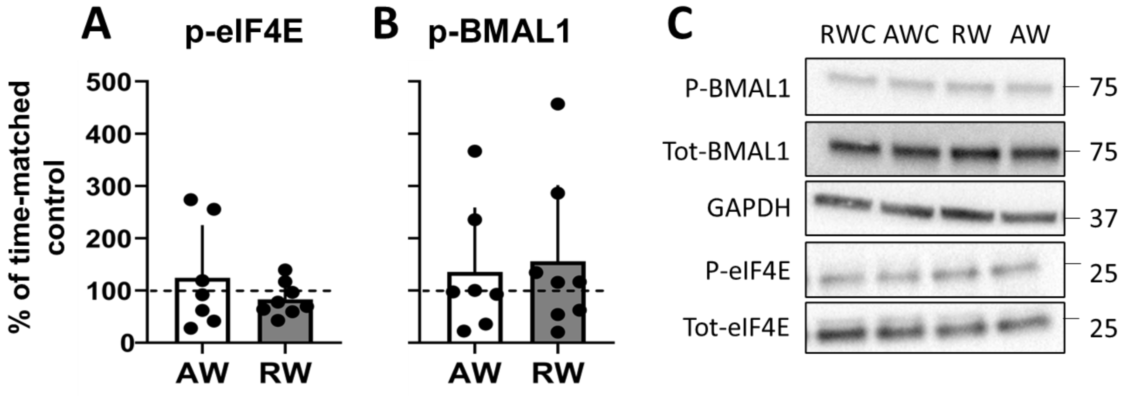

Fig. S1. Expression of inputs of promoters of cap-dependent translation initiation in prefrontal cortex following 3 days active work (AW, collected at zeitgeber (ZT0) and rest work (RW, collected at ZT12). (A-B) western blot analysis. (C) Representative immunoblots for (A-B). Full-length blots/gels are presented in Supplementary Figure S3. Blots normalized to GAPDH in the corresponding immunoblot. Phospho-protein normalized to total protein in the corresponding immunoblot. Quantification of immunoblots are expressed as percentage change relative to time-matched undisturbed control (AWC collected at ZT0, RWC collected at ZT12; normalized to 100%). Plots show mean ±SEM, with scatter plot overlaid. N = 7-8/group.

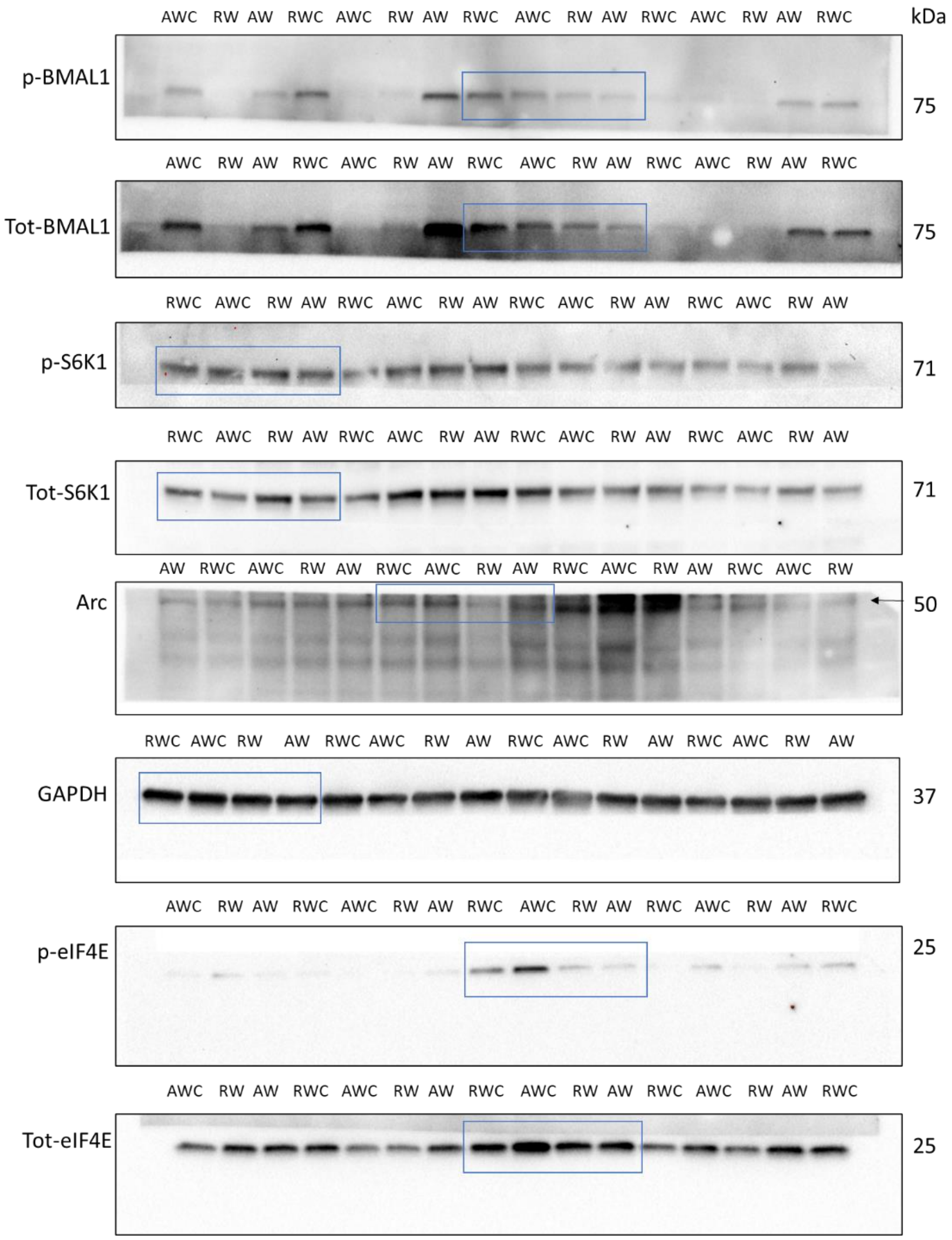

Fig. S2. Representative immunoblots for figure 3. Membranes were cut to allow for probing for different proteins. Blue squares indicate the part of the original image that was cropped.

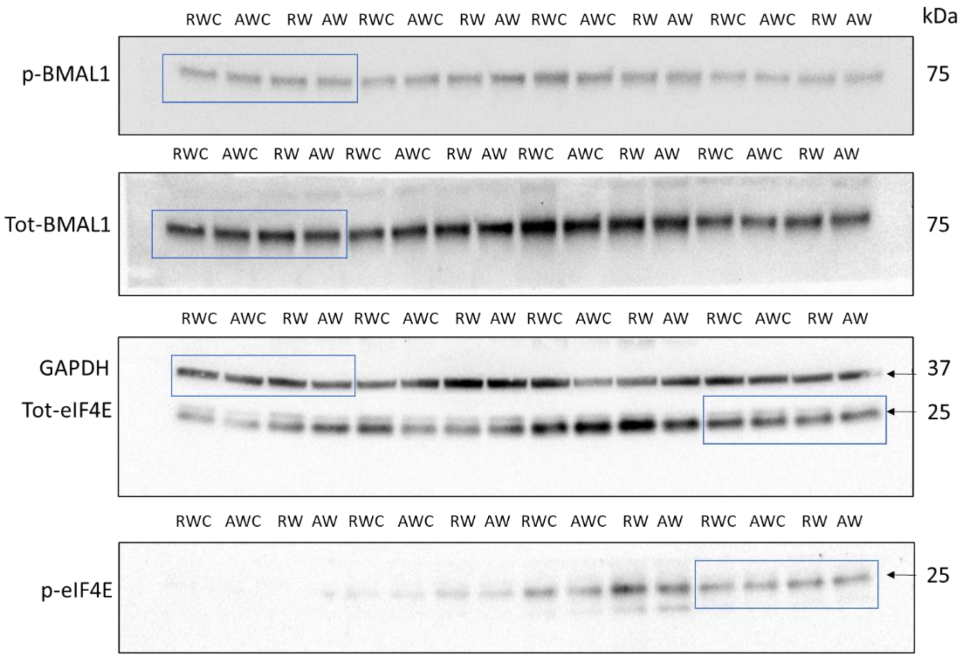

Fig. S3. Representative immunoblots for figure S1. Membranes were cut to allow for probing for different proteins. Blue squares indicate the part of the original image that was cropped.

Table S1: Hierarchical regression analyses for predictors of Morris Water Maze (MWM) performance following simulated shift work, test for contribution of SWE in NREM sleep.

	**MWM latency to platform**
**Step 1, work condition**	$F_{(1,11)} = 3.96$, $p = .07$
AW/RW	
R^2	.23
Adj. R^2	.20
**Step 2, daily rhythm dynamics**	$F_{(3,9)} = 2.52$, $p = .12$
Amplitude change	
REM sleep latency	
R^2	.46
Adj. R^2 (change)	.28 (.08)
**Step 3, sleep intensity**	$F_{(4,7)} = 1.40$, $p = .33$
SWE in NREM sleep	
R^2	.46
Adj. R^2 (change)	.13 (-.15)

AW active work, RW rest work, SWE slow wave energy, REM rapid eye movement sleep, NREM non rapid eye movement sleep

Table S2: Hierarchical regression analyses for predictors of Morris Water Maze (MWM) performance following simulated shift work, test for contribution of 72h cumulated sleep parameters.

	**MWM latency to platform**
**Step 1, work condition**	$F_{(1,11)} = 3.96$, $p = .07$
AW/RW	
R^2	.23
Adj. R^2	.20
**Step 2, daily rhythm dynamics**	$F_{(3,9)} = 2.52$, $p = .12$
Amplitude change	
REM sleep latency	
R^2	.46
Adj. R^2 (change)	.28 (.08)
**Model 3, cumulated sleep**	$F_{(5,6)} = 1.22$, $p = .39$
NREM sleep time	
REM sleep time	
R^2	.47
Adj. R^2 (change)	.08 (-.20)

AW active work, RW rest work, REM rapid eye movement sleep, NREM non rapid eye movement sleep

Table S3: Hierarchical regression analyses for predictors of prefrontal cortex protein expression in inputs following simulated shift work.

	p-eIF4E	p-BMAL1
**Step 1, work condition**	$F_{(1,10)}$ = 1.24,	$F_{(1,10)}$ = 0.1,
	p = .29	p = .76
AW[1]/RW[2]		
R^2	.11	.01
Adj. R^2	.02	-.09
**Step 2, daily rhythm**	$F_{(3,8)}$ = 1.8,	$F_{(3,8)}$ = 0.35,
**dynamics**	p = .22	p = .79
Amplitude change		
REM sleep latency		
R^2	.40	.11
Adj. R^2 (change)	.18 (.16)	-.22 (-.13)
**Step 3, sleep drive**	$F_{(5,5)}$ = 3.33,	$F_{(5,5)}$ = 0.84,
	p = .11	p = .57
Duration of NREM sleep bouts		
SWE in QW		
R^2	.77	.46
Adj. R^2 (change)	.54 (.36)	-.09 (.13)
**Model 4, serum**	$F_{(6,4)}$ = 4.31,	$F_{(6,4)}$ = 0.7,
**corticosterone**	p = .09	p = .67
R^2	.87	.51
Adj. R^2 (change)	.67 (.13)	-.22 (-.13)

AW active work, RW rest work, SWE slow wave energy, QW quiet wakefulness, REM rapid eye movement sleep, NREM non rapid eye movement sleep

Table S4: Hierarchical regression analyses for predictors of prefrontal cortex protein expression following simulated shift work, test for contribution of SWE in NREM sleep.

	p-eIF4E	p-BMAL1	p-S6K1	Arc
**Step 1, work condition**	$F_{(1,7)} = 3.98$, $p = .09$	$F_{(1,11)} = 8.68$, $p = .013$	$F_{(1,11)} = 72.39$, $p < .001$	$F_{(1,8)} = 1.76$, $p = .22$
AW/RW				
R^2	.36	.44	.87	.18
Adj. R^2	.27	.39	.86	.08
**Step 2, daily rhythm dynamics**	$F_{(3,5)} = 2.59$, $p = .17$	$F_{(3,9)} = 5.07$, $p = .03$	$F_{(3,9)} = 22.58$, $p < .001$	$F_{(3,6)} = 0.49$, $p = .70$
Amplitude change				
REM sleep latency				
R^2	.61	.63	.88	.20
Adj. R^2 (change)	.37 (.10)	.50 (.11)	.84 (-.02)	-.20 (-.28)
**Step 3, sleep intensity**	$F_{(4,3)} = 1.27$, $p = .44$	$F_{(4,7)} = 2.8$, $p = .111$	$F_{(4,7)} = 13.91$, $p = .002$	$F_{(4,4)} = 0.77$, $p = .60$
SWE in NREM sleep				
R^2	.63	.63	.89	.43
Adj. R^2 (change)	.13 (-.24)	.40 (-.10)	.82 (-.02)	-.13 (.07)

AW active work, RW rest work, SWE slow wave energy, REM rapid eye movement sleep, NREM non rapid eye movement sleep

Table S5: Hierarchical regression analyses for predictors of prefrontal cortex protein expression following simulated shift work, test for contribution of 72h cumulated sleep parameters.

	p-eIF4E	p-BMAL1	p-S6K1	Arc
**Step 1, work condition**	$F_{(1,7)} = 3.98$, $p = .09$	$F_{(1,11)} = 8.68$, $p = .013$	$F_{(1,11)} = 72.39$, $p < .001$	$F_{(1,8)} = 1.76$, $p = .22$
AW/RW				
R^2	.36	.44	.87	.18
Adj. R^2	.27	.39	.86	.08
**Step 2, daily rhythm dynamics**	$F_{(3,5)} = 2.59$, $p = .17$	$F_{(3,9)} = 5.07$, $p = .03$	$F_{(3,9)} = 22.58$, $p < .001$	$F_{(3,6)} = 0.49$, $p = .70$
Amplitude change				
REM sleep latency				
R^2	.61	.63	.88	.20
Adj. R^2 (change)	.37 (.10)	.50 (.11)	.84 (-.02)	-.20 (-.28)
**Step 3, cumulated sleep**	$F_{(5,3)} = 0.98$, $p = .54$	$F_{(5,7)} = 3.48$, $p = .07$	$F_{(5,7)} = 12.4$, $p = .002$	$F_{(5,4)} = 0.32$, $p = .88$
NREM sleep time				
REM sleep time				
R^2	.62	.71	.90	.28
Adj. R^2 (change)	-.02 (-.39)	.51 (.01)	.83 (-.01)	-.61 (-.41)

AW active work, RW rest work, REM rapid eye movement sleep, NREM non rapid eye movement sleep
